# Natural Occurrence of *Alternaria* Toxins in Wheat-Based Products and Their Dietary Exposure in China

**DOI:** 10.1371/journal.pone.0132019

**Published:** 2015-06-29

**Authors:** Kai Zhao, Bing Shao, Dajin Yang, Fengqin Li, Jianghui Zhu

**Affiliations:** 1 National Institute for Nutrition and Health, Chinese Center for Disease Control and Prevention, Beijing, People’s Republic of China; 2 Key Laboratory of Food Safety Risk Assessment, China National Center for Food Safety Risk Assessment, Beijing, People’s Republic of China; 3 Department of Central Laboratory, Beijing Municipal Center for Disease Control and Prevention, Beijing, People’s Republic of China; Institute of Genetics and Developmental Biology, CHINA

## Abstract

A total of 181 wheat flour and 142 wheat-based foods including dried noodle, steamed bread and bread collected in China were analyzed for alternariol (AOH), alternariol monomethyl ether (AME), tentoxin (TEN) and tenuazonic acid (TeA) by ultra-performance liquid chromatography-electrospray ionization-tandem mass spectrometry. TeA was the predominant toxin found in 99.4% wheat flour samples at levels ranging from 1.76 μg/kg to 520 μg/kg. TEN was another *Alternaria* toxin frequently detected in wheat flour samples (97.2%) at levels between 2.72 μg/kg and 129 μg/kg. AOH and AME were detected in 11 (6.1%) samples at levels ranging from 16.0 μg/kg to 98.7 μg/kg (AOH) and in 165 (91.2%) samples with a range between 0.320 μg/kg and 61.8 μg/kg (AME). AOH was quantified at higher levels than AME with the ratio of AOH/AME ranging from 1.0 to 3.7. Significant linear regressions of correlation in toxin concentrations were observed between AOH and AME, AME and TeA, TEN and TeA, AOH+AME and TeA. At an average and 95th percentile, dietary exposure to AOH and AME in the Chinese general population and different age subgroups exceeded the relevant threshold value of toxicological concern (TTC), with the highest exposure found in children which deserves human health concern. TEN and TeA seem unlikely to be health concerns for the Chinese via wheat-based products but attention should be paid to synergistic or additive effects of TeA with AOH, AME, TEN and a further assessment will be performed once more data on toxicity-guided fractionation of the four toxins are available. It is necessary to conduct a systemic surveillance of *Alternaria* toxins in raw and processed foods in order to provide the scientific basis for making regulations on these toxins in China.

## Introduction

Cereal grains are frequently infected by species of *Alternaria* that are capable of producing a number ofmycotoxins. The most important *Alternaria* toxins that have been chemically characterized and have documented toxicity to animals can be grouped into three different structural classes: dibenzopyrone derivatives as in alternariol (AOH), alternariol monomethyl ether (AME), and altenuene (ALT); a tetramic acid derivative tenuazonic acid (TeA); and the perylene derivatives altertoxins I, II and III (ATX I, II and III) [1,2]. These toxins have been shown to have harmful effects in animals, including cytotoxic, fetotoxic and teratogenic activities [3]. They are also mutagenic, clastogenic and estrogenic in microbial and mammalian cell systems and tumorigenic in rats [4–7]. In addition, consumption of cereals invaded by *Alternaria* species and contaminated with associated mycotoxins was related to risk of human esophageal cancer in China [8].

Risk assessments relating to food safety are frequently hampered by the lack of quantitative data. According to European Food Safety Authority (EFSA), one of the contributions of dietary exposure to *Alternaria* toxins is made by grain and grain-based products, especially wheat products [9]. Wheat is an important crop in the north part of China and wheat-based products are the staple foods for human consumption in these areas. Although *Alternaria* toxin is a ubiquitous contaminant of several classes of commodities, contamination of wheat likely poses the greatest health risk to humans in China. This is due primarily to the importance of this commodity as a food source throughout the country, especially in esophageal cancer hyperendemic regions, where *Alternaria* toxin-contaminated wheat is still a staple food for human consumption, as well as to the fact that wheat is conducive to *Alternaria* invasion and toxin production. Additionally, although contamination of Chinese weathered wheat by *Alternaria* toxins has been described, quantitative data on contamination by AOH, AME and TeA in wheat products for human consumption are still limited [10]. A potential risk of human dietary exposure to *Alternaria* toxins has never been demonstrated and no regulations on *Alternaria* toxins in foods and feeds are available in China so far. In order to develop strategies to reduce risk from these contaminants, the objective of this study was to evaluate the natural occurrence of *Alternaria* mycotoxins in Chinese wheat-based food products, and to conduct a preliminary risk assessment based on both the contamination data for *Alternaria* toxins in wheat-based products and food consumption data of Chinese populations. The results obtained in our study will be expected to contribute to further research on conducting a systemic surveillance of *Alternaria* toxins in raw and processed foods and to develop strategies for reducing risk from these contaminants.

## Materials and Methods

### Reagents and Chemicals

Methanol and acetonitrile used for sample extraction and toxin separation were of HPLC grade (Dikma Pure, Richmond Hill, GA). Both ammonium bicarbonate (Sigma-Aldrich, St. Louis, MO) and formic acid (≥ 95% purity) (Fluka, Steinheim, Germany) were analytical grade. Pure water was obtained from a Millipore Milli-Q System (Millipore, Bedford, MA). Standards for alternariol (purity > 98%), alternariol monomethyl ether (purity > 98%), tentoxin (purity > 99%) and tenuazonic acid (purity > 99%) were purchased from Fermenteck Ltd. (Fermenteck, Jerusalem, Israel). All experimental practice followed Environmental Health Safety Guidelines for the use of chemicals authorized by China National Center for Food Safety Risk Assessment.

### Samples

A total of 181 packaged wheat flour samples originating from the 2013 crop were randomly collected by visiting supermarkets and agricultural trade markets in Henan, Shandong, Anhui, and Jilin provinces and in Beijing, which are the most important wheat-producing regions with yearly wheat outputs accounting for about 70% of the total in China. Wheat constitutes about 80% of the cereal crops and wheat-based foods such as steamed bread, noodles, and pancakes, which are the staple foods for human consumption in these areas. In Beijing, 142 wheat—based products including dried noodles (*n* = 52), bread (*n* = 50) and steamed bread (*n* = 40) were collected from supermarkets and agricultural trade markets. Since Beijing is the capital of China, the major brand of dried noodles produced all over China can be found on the Beijing market. Hence these samples are representative. All samples were kept at either cool and dry conditions (dried noodle) or at 4°C (bread and steamed bread) until analysis.

With regard to reducing the wheat flour sample to a subsample for analysis, three incremental samples were drawn from each of the top, middle and bottom layers of the containers. Multiple subsamples (ca. 500 g) were pooled, mixed thoroughly and quartered. Opposite quarters were rejected and the remainder re-mixed thoroughly to obtain a final reduced sample of 250 g. Half of the entire composite was combined, finely ground for wheat-based foods, and a 5 g test portion was taken for analysis. All ground samples were kept in ziplock plastic bags at -30°C prior to analysis.

### Toxin analysis

All wheat flour and wheat-based food samples were analyzed for AOH, AME, TEN and TeA based on methods described previously with some modification [11, 12]. In brief, a finely ground sample (5g) was extracted with 20 ml acetonitrile-water-methanol (45:45:10, v/v/v), sonicated for 30 min followed by centrifuging for 10 min at 7000 rpm. A 5 ml portion of supernatant was diluted 5 times with distilled water, the pH was adjusted to a range between 3 and 4 with formic acid, and chromatographed on a HLB solid phase extraction (SPE) cartridge (Waters, Switzerland). The four *Alternaria* toxins were eluted with 5 ml methanol followed by 5 ml acetonitrile. Both eluates were combined, evaporated to dryness and dissolved in 1 ml methanol-water (10:90, v/v) containing 0.5 mmol/L ammonium bicarbonate and centrifuged for 5 min at 10000 rpm. The supernatant was analyzed for the four *Alternaria* toxins by UPLC-MS/MS based under the conditions described by Zhao et al. [11]. Matrix-matched calibration was used for quantification of the four mycotoxins in wheat flour and wheat-based products. Mean recoveries, in which the matrix effect was compensated for, were in the ranges 82.9–115.5% (TeA), 70.1–110.7% (AOH), 71.8–101.2% (TEN) and 78.8–102.9% (AME), as determined from six parallel analyses of blank wheat samples spiked with 5–25 μg/kg (TeA), 20–100 μg/kg (AOH), 1–5 μg/kg (TEN) and 0.25–1.25 μg/kg (AME) with the respective coefficient of variation (CV) of 9.9–13.1% for TeA, 4.4–16.5% for AOH, 10.7–12.3% for TEN, and 6.6–12.4% for AME, respectively. The limits of detection for TeA, AOH, TEN and AME were 2 μg/kg, 8 μg/kg, 0.8 μg/kg and 0.2 μg/kg, respectively.

### Food consumption data

Food consumption data were provided by the China national nutrient and health survey of 2002 using three consecutive 24 hours dietary recall from face-to-face interviews, including one weekend. Consumption data from a total of 68,959 subjects aged 2–100 years old were employed.

### Data analysis

*Kruskal-Wallis* H Test was employed for statistical analysis in comparison of toxin concentrations in different food matrices (steamed bread, dried noodles, bread, wheat flour) followed by applying *Mann-Whitney* U Test for further analysis if there was a significant difference in toxin concentration between any both food matrices. The correlations in concentrations between any two toxins were examined by simple *Spearman* correlation analysis.

### Calculation of dietary exposure to *Alternaria* toxins by the Chinese populations

Data on natural occurrence of the four toxins in wheat-based products were processed according to the principles recommended by the World Health Organization (WHO) and EFSA [13, 14]. A lower bound-upper bound (LB-UB) approach was employed for AOH. The lower bound assigns a value of zero for left-censored results and the upper bound assigns the value of LOD to results below LOD. A substitution method was used for TeA, TEN and AME, in which left-censored data were assigned the value of half LOD. The threshold of toxicological concern (TTC) approach was employed to assess the relative levels of concern of these four mycotoxins to human health as recommended by the literatures [15, 16]. The mean, median, 97.5 percentile and the maximum dietary exposures were derived for the general and subgroups of population, including children (2–6 years), adolescent (7–17 years), adults (18–59 years) and the elderly (≥ 60 years). Individual food consumption data were combined with the mean occurrence values of each toxin in order to provide mean and high percentile (95th percentile) exposure estimations. The mean dietary exposure (average consumption in different age populations) and the high dietary exposure (95th percentile food consumption) to *Alternaria* toxins were calculated separately.

## Results and Discussion

### Natural occurrence of *Alternaria* mycotoxins in wheat flour and wheat-based foods

Natural occurrence of the four *Alternaria* toxins in Chinese wheat-based products is given in Table 1. In the supplemental materials, concentrations of four *Alternaria* toxins in all wheat-based food samples are presented (S1 Table). TeA was the predominant *Alternaria* toxin in terms of either frequency or concentration, and it was detected in 99.4% (180/181) wheat flour samples at levels ranging from 1.76 μg/kg to 520 μg/kg (mean = 79.80 μg/kg). Twelve samples (6.6%) were contaminated with TeA at a concentration higher than 200 μg/kg with a maximum of 520 μg/kg, 45 (24.9%) samples had TeA levels between 100 μg/kg and 200 μg/kg, and 123 (68.0%) samples had TeA below 100 μg/kg. These results were similar to those reported in Germany by Siegel et al [17] but the positive rate obtained in the present study was much higher than that reported in Germany by Müller et al [18]. TEN was another *Alternaria* toxin frequently detected in wheat flour samples. It was quantified in 176 (97.2%, 176/181) wheat flour samples with a range between 2.72 μg/kg and 129 μg/kg (mean: 27.1 μg/kg). Sixteen samples (8.8%, 16/181) were positive for TEN at a concentration higher than 50 μg/kg, 134 (74.0%) samples were between 10 μg/kg and 50 μg/kg, and 26 (14.4%) samples were below 10 μg/kg. Of 181 wheat flour samples analyzed, 11 (6.1%) samples were positive for AOH at levels ranging from 16.0 μg/kg to 98.7 μg/kg (mean = 30.2 μg/kg) and 165 (91.2%) samples contained AME with a range between 0.320 μg/kg and 61.8 μg/kg (mean = 3.77 μg/kg). AOH was found at higher levels than AME with the ratio of AOH/AME ranging from 1.0 to 3.7 (average = 1.9) in 11 AOH positive samples. AME was found more frequently than AOH. A total of 13 samples (7.2%) were contaminated with AME at a concentration higher than 10 μg/kg with a maximum of 50 μg/kg, 18 (9.9%) samples were between 5 μg/kg and 10 μg/kg, and 134 (74.0%) samples were below 5 μg/kg.

**Table 1:** Concentrations of TeA, AOH, TEN, AME in wheat flour and wheat-based food samples.

Samples	Toxins	Positive (%)	Range (μg/kg)	Mean (μg/kg)	Median (μg/kg)	SD (μg/kg)#
Wheat flour (*n* = 181)	TeA*	180 (99.4)	1.76–520	88.4	77.5	66.7
	AOH	11 (6.1)	16.0–98.7	30.2	24.0	23.6
	TEN*	176 (97.2)	2.72–129	27.1	23.4	19.2
	AME*	165 (91.2)	0.320–61.8	3.77	1.92	6.40
Dried noodle (*n* = 52)	TeA	50 (96.2)	4.86–158	47.6	32.9	39.4
	AOH	3 (5.8)	9.59–11.8	10.7	10.7	1.09
	TEN	46 (88.5)	2.25–32.3	13.9	12.8	8.57
	AME	32 (61.5)	0.18–4.10	1.17	0.724	1.07
Bread (*n* = 50)	TeA	49 (98.0)	1.95–38.2	11.7	8.81	9.21
	AOH	1 (2.0)	9.98	-	-	-
	TEN	41 (82.0)	3.13–27.2	8.37	7.71	4.82
	AME	22 (44.0)	0.18–6.49	1.07	0.561	1.39
Steamed bread (*n* = 40)	TeA	40 (100)	6.56–46.3	21.2	19.5	9.66
	AOH	0	-	-	-	-
	TEN	40 (100)	2.46–31.6	10.7	7.47	8.15
	AME	29 (72.5)	0.210–1.41	0.640	0.620	0.330

*Toxin concentrations in wheat flour were significantly higher than those in wheat-based foods (*P*<0.01).

^#^ *SD*: standard deviation.

With regard to the contamination of the four *Alternaria* toxins in wheat-based foods, TeA was also the toxin most frequently found in dried noodles, bread and steamed bread samples with a frequency of 96.2% (50/52), 98.0% (49/50), 100% (40/40), respectively and average concentrations of 47.6 μg/kg, 11.7 μg/kg and 21.2 μg/kg, respectively, much lower than those in wheat flour. The mean and median values of TeA in dried noodle, bread and steamed bread samples were similar and indicated a high portion of wheat-based food samples contaminated with TeA (Table 1). Similarly, high frequencies of TEN contamination were also found in dried noodles (88.5%), bread (82.0%) and steamed bread (100%) samples, with a maximum level of 32.3 μg/kg in a dried noodle sample. Regarding the dibenzopyrone derivatives, AME was found in high frequencies (from 44.0% to 72.5%) but at very low levels (the highest value was 6.49 μg/kg in a bread sample). Only 3 dried noodle and 1 bread sample were positive for AOH with the concentration up to 11.8 μg/kg.

Notably, the four *Alternaria* toxins in two wheat-based foods (bread and steamed bread) were found at a considerable level, although lower than those in wheat flour and unheated food (dried noodle) (*P*<0.01). These mainly due to the fact that the lot of wheat flour used for preparation of dried noodles, steamed bread and bread is different from those of wheat flour we analyzed in the present study. Additionally, these also revealed that *Alternaria* toxins are stable to some extent to heat during food processing since the bread is baked at a temperature higher than 200°C for more than 30 min, while steamed bread would be heated at 100°C for more than 15 min. The results also illustrated that the toxins might be present in other processed wheat products due to the limitations of the current industrial procedures to eliminate the rotting grains completely. Little information is available on the behavior of *Alternaria* toxins in food during the storage and processing but there are some indications that concentrations of *Alternaria* toxins may increase under favorable conditions. So far, surveys on the fate of *Alternaria* toxins during food processing in Chinese style have rarely been reported and should be conducted in the near future.

### Distribution of the four *Alternaria* toxins in wheat flour from different regions

The distribution of the four *Alternaria* toxins in different provinces is given in Table 2. The levels of the four *Alternaria* toxins in wheat flour varied geographically, with high concentrations found in south China (Anhui province), where high temperature, humidity, and precipitation are observed, in comparison with concntrations from central (Henan province) and north China (Shandong, Jilin province and Beijing). TeA was detected in 100% samples from Jilin province where average (51.5 μg/kg) and median (41.5 μg/kg) concentrations were significantly lower than those from Anhui (Mann-Whitney U test, *P* = 0.000), Henan (*P* = 0.000), Shandong (*P* = 0.002) and Beijing (*P* = 0.046). In addition, TeA was also found at higher levels (mean:110 μg/kg, median:100 μg/kg) in samples from Henan than those from Shandong province (mean: 78.6 μg/kg, median: 67.8 μg/kg, *P* = 0.022). No differences in TeA concentration between samples from Shandong and Anhui, Beijing and Henan, Beijing and Shandong, Beijing and Anhui, Henan and Anhui provinces were found. Concerning the other three *Alternaria* toxins, the geographical distribution in concentrations of either average or median of TEN and AME were almost similar to those of TeA. The prevalence of both toxins in samples from five regions were in the range of 85.7% to 96.0% for TEN and from 78.6% to 96.0% for AME. The concentrations of TEN and AME in samples from Anhui (Mann-Whitney U test, *P* = 0.002 for TEN and *P* = 0.000 for AME), Henan (*P* = 0.000 for TEN and *P* = 0.000 for AME) and Shandong (*P* = 0.019 for TEN and *P* = 0.030 for AME) were significantly higher than those from Jilin, a northeast part of China with lower temperature, lower average annual precipitation, and drought. Additionally, wheat flour samples from Henan province were heavily contaminated with TEN, compared with those from Beijing (*P* = 0.031) and Shandong (*P* = 0.000) province. Also much higher levels of AME were determined in samples from Henan than Shandong (*P* = 0.001), from Anhui than Shandong (*P* = 0.000), from Beijing than Shandong (*P* = 0.013), and from Beijing than Jilin (*P* = 0.004). The highest levels of TeA, TEN, AME and AOH, either the mean, median or the maximum, were found in samples from Anhui province. These verified that the production of *Alternaria* toxins was closely related to weather conditions. With regard to TEN, the highest average concentration was quantified in samples from Anhui (35.4 μg/kg) followed by Henan (34.2 μg/kg), Beijing (23.8 μg/kg), Shandong (22.9 μg/kg) and Jilin (19.7 μg/kg). As for AME, the levels of this toxin in wheat flour samples were much lower than for TeA and TEN, with the highest level up to 61.8 μg/kg and an average of 9.26 μg/kg in samples from Anhui province. AOH was detected in samples from Henan (4/44, 9.1%) and Anhui (7/24, 29.2%) provinces at low levels with the maximum of 98.7 μg/kg. All samples from Shandong, Beijing and Jilin were negative for AOH.

**Table 2:** Concentrations of TeA, AOH, TEN, and AME in wheat flour samples from different provinces.

Toxins	Sampling sites	*n*	Positive (%)	Range (μg/kg)	Mean (μg/kg)	Median (μg/kg)	*SD* (μg/kg)
TeA	Henan	44	43 (97.7)	8.96–265	110	100	66.6
	Shandong	60	60 (100)	21.4–228	78.6	67.8	44.4
	Anhui	24	24 (100)	4.00–520	118	87.3	108
	Beijing	25	25 (100)	6.08–265	87.5	82.2	66.4
	Jilin*	28	28 (100)	3.36–155	51.5	41.5	36.5
AOH	Henan	44	4 (9.1)	16.0–19.2	17.2	16.9	1.38
	Shandong	60	0	-	-	-	-
	Anhui	24	7 (29.2)	24.0–98.7	37.6	24.2	27.5
	Beijing	25	0	-	-	-	-
	Jilin	28	0	-	-	-	-
TEN	Henan	44	42 (95.5)	4.64–85.8	35.2	30.7	19.4
	Shandong	60	60 (100)	5.28–75.5	22.9	18.8	13.4
	Anhui	24	23 (95.8)	11.2–98.9	35.4	27.8	23.1
	Beijing	25	24 (96.0)	4.62–61.8	23.8	21.4	15.5
	Jilin*	28	24 (85.7)	2.72–129	19.7	15.5	23.2
AME	Henan	44	38 (86.4)	0.80–15.7	4.18	3.20	3.55
	Shandong	60	59 (98.3)	0.32–18.6	1.84	1.28	2.41
	Anhui	24	23 (95.8)	0.80–61.8	9.26	4.16	14.1
	Beijing	25	24 (96.0)	0.32–12.6	3.76	2.56	3.39
	Jilin*	28	22 (78.6)	0.32–18.9	2.42	0.96	4.02

*TeA, TEN and AME concentrations in samples from Jilin were lower than those in samples from other 4 sites (*P*<0.05).

### Co-occurrence of the four *Alternaria* toxins in wheat flour

**Fig 1:**
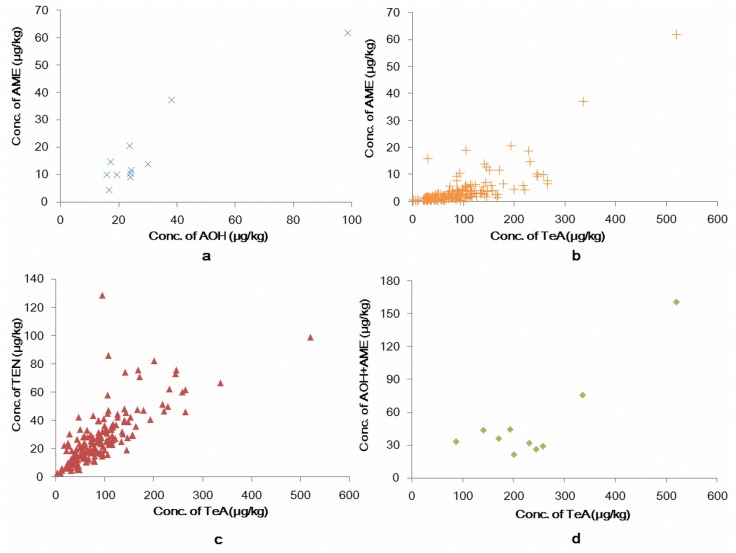
Correlation of concentrations of the four *Alternaria* mycotoxins in wheat flour. a: AOH vs AME, b: TeA vs AME, c: TeA vs TEN, d: TeA vs (AOH + AME).

With regard to the co-occurrence of *Alternaria* toxins in wheat flour samples, TEN and TeA, AME and TeA, and AOH and AME occurred together in 176 (97.2%, 176/181), 165 (91.2%, 165/181) and 11 (6.1%, 11/181) samples, respectively, and significant linear regressions of correlation in toxin concentration between AOH and AME (*r* = 0.877, *P*<0.01), TeA and AME (*r* = 0.757, *P*<0.01), TEN and TeA (*r* = 0.747, *P*<0.01) were observed (Fig 1). Moreover, the total dibenzopyrone derivatives (AOH + AME) and TeA (*r* = 0.860, *P*<0.01) were detected together in 10 samples (9.1%). This indicated the co-production of these toxins on wheat grains by *Alternaria* species in fields and the results were similar to those in weathered wheat in China reported by Li and Yoshizawa [19].

### Dietary exposure to the four *Alternaria* toxins in Chinese populations

There are no risk assessments for *Alternaria* toxins in food and feed carried out at international level, except brief assessment by EFSA [19]. To our knowledge, this is the first report on dietary exposure to *Alternaria* toxins in China.

AOH and AME were both strongly mutagenic to some bacterial cells [20–21] and could induce DNA strand breaks [22] as an inhibitor of DNA topoisomerase I and II [23]. The threshold value for both *Alternaria* toxins was 2.5 ng/kg body weight (0.15 μg/person) per day derived from the TTC decision tree [15]. Considering the limited occurrence data, an estimation of dietary exposure was conducted based on the TTC value. The dietary exposure in the general population was estimated to be in the following ranges: AOH: 3.56 (LB)- 24.0 ng/kg body weight per day (UB) for the average and 17.0 (LB)- 102 ng/kg body weight per day (UB) for the 97.5^th^ percentile, which exceed the TTC value up to 9.6 times higher for the average and 40.8 times higher for the 97.5^th^ percentile. As for different age subgroups, the average dietary exposures are all above the TTC value with the highest found in children: 2.4 and 15.6 times higher than the TTC value for LB and UB, respectively. At the 97.5^th^ percentile, exposure was estimated at 4.9 (LB) and 4.3 (UB) times higher for the general population, 5.5 (LB) and 4.5 (UB) times higher for children aged 2–6, 4.9 (LB) and 4.2 (UB) times higher for adolescents aged 7–17, 4.7 (LB) and 4.0 (UB) times higher for adults aged 18–59, and 5.0 (LB) and 4.2 (UB) times higher for the elderly aged 60 and above, compared to the corresponding mean dietary exposure (Table 3).

**Table 3:** Estimation of exposure to the four *Alternaria* toxins in Chinese populations (ng/kg body weight per day).

Population	Statistics	AOH (LB)	AOH (UB)	AME	TEN	TeA
General	mean	3.56	24.0	6.09	54.5	175
	P50	1.30	13.1	1.71	23.2	75.0
	P97.5	17.6	102	32.4	257	840
Children*	mean	5.90	39.1	10.2	90.1	292
	P50	1.90	20.1	2.38	35.2	115
	P97.5	32.2	175	58.3	469	1537
Adolescent^#^	mean	4.72	31.0	8.17	71.9	232
	P50	1.58	16.4	2.05	28.6	92.9
	P97.5	23.0	131	42.7	335	1106
Adults	mean	3.24	21.9	5.52	49.7	160
	P50	1.27	12.5	1.68	22.5	73.0
	P97.5	15.1	87.6	28.2	221	727
Elderly	mean	2.67	18.6	4.50	41.3	131
	P50	1.11	11.2	1.40	19.6	61.6
	P97.5	13.3	78.1	24.7	195	640

* Children dietary exposures at mean, P50 and P97.5 levels were higher than other age subgroups (*P*<0.05).

^#^ Adolescence dietary exposures at mean, P50, P97.5 levels were higher than those in adults and elderlies (*P*<0.05).

The average and 97.5^th^ percentile dietary exposures to AME were 6.09 ng/kg body weight per day and 32.4 ng/kg body weight per day, respectively, both of which exceed the TTC value. Moreover, all average and 97.5^th^ percentile dietary exposures in different age subgroups exceed the TTC value, with the highest found in children. The dietary exposure to AME in people with high consumption of wheat-based foods would be several times higher than the TTC value. The estimation of exposure to either AOH or AME indicates the need for additional toxicity data to assess the potential health risk, especially for the children and the populations with high consumption.

TeA and TEN are classified as Cramer structural class III by software tools Toxtree (v2.5) [16]. Therefore, the value of 1500 ng/kg body weight per day (90 μg/person) was used for both toxins in risk assessment [15]. For TEN, dietary intake ranged from 41.3 to 90.1 ng/kg body weight per day for the average and from 195 to 469 ng/kg body weight per day for the 97.5^th^ percentile in different age subgroups, which are much lower than the TTC value. For TeA, the general population intakes were 175 ng/kg body weight for the average and 840 ng/kg body weightw for the 97.5^th^ percentile, much higher than those reported by EFSA (mean ≤ 13 ng/kg body weight per day) but lower than the TTC value [16]. Hence, TEN and TeA seem unlikely to be human health concerns. However, the 97.5^th^ percentile dietary exposure to TeA in children aged 2–6 years old was 1537 ng/kg body weight per day, slightly higher than the TTC value (Table 3). Considering the synergistic or additive effects among AOH, AME, TEN and TeA might be not ruled out, further assessment should be carried out once more data on toxicity-guided fractionation of the four toxins available. So at either an average or the 97.5^th^ percentile, dietary exposure of different age subgroups to all four *Alternaria* toxins were in the following order: children > adolescent > adults > elderly.

### Uncertainty

Some uncertainties in assessment of dietary exposure to the four *Alternaria* toxins should be acknowledged. Firstly, limited toxin occurrence data in wheat-based products are not representative of all food commodities that *Alternaria* toxins could contaminate. Secondly, food consumption data used in this study were from the latest version of the China National Nutrition and Health Survey conducted in 2002, and consumption patterns may have changed during the 13-year long interval. Thirdly, limited toxicity data for the four *Alternaria* toxins, and the use of LB/UB bound occurrence data for AOH in risk assessment might have led to an over- or under-estimation of the human exposure. Thirdly, the synergistic or additive effects among AOH, AME, TEN and TeA are not considered. Finally, only exposure through food was considered and this does not account for additional exposure pathways such as house dust.

## Conclusions

To the best of our knowledge, this is the first report on natural occurrence of the four *Alternaria* toxins in wheat-based products for human consumption and estimation of their implications for public health risk in China. TeA, whether frequency or concentration, was the predominant *Alternaria* toxin detected in wheat flour and wheat-based foods, followed by TEN, AOH and AME. The levels of the four *Alternaria* toxins in wheat flour and wheat-based food samples varied geographically. Toxin concentrations in samples from south China, where high temperature, humidity, and precipitation are observed, were higher than those from central and north China. Significant linear regressions of correlation in toxin concentrations were observed between TEN and TeA, AME and TeA, and between AOH and AME. On average, dietary exposure to AOH and AME in the Chinese general population and different age subgroups exceeded the relevant TTC values and deserve human health concern, with the highest exposure found in children. Although TEN andTeA seem unlikely to be health concerns according to the results of this evaluation, attention should be paid to their synergistic or additive effects with AOH and AME and perform a further assessment once more data on toxicity-guided fractionation of the four toxins available. Dietary exposure of children to the four *Alternaria* toxins was higher than for any other subgroups in the present study and should be of concern.

## Supporting Information

S1 Table Concentrations of Four *Alternaria* toxins in Wheat-based Food Samples. (DOCX)
