# Supplementary material for: Natural Occurrence of Alternaria Toxins in Wheat-Based Products and Their Dietary Exposure in China
Source: PLoS One. 2015 Jun 29;10(6):e0132019. doi: 10.1371/journal.pone.0132019 (PMC4487895; doi:10.1371/journal.pone.0132019)
Supplement: S1 Table — (DOCX) [file pone.0132019.s001.docx]

**S1 Table. Concentrations of Four *Alternaria* toxins in Wheat-based Food Samples**

| Number | food category | Collecting sites | TeA（μg/kg） | AOH（μg/kg） | TEN（μg/kg） | AME（μg/kg） |
| --- | --- | --- | --- | --- | --- | --- |
| 1 | Wheat flour | Henan | <LOD | <LOD | <LOD | <LOD |
| 2 | Wheat flour | Anhui | 519.68 | 98.72 | 98.88 | 61.76 |
| 3 | Wheat flour | Anhui | 335.68 | 38.24 | 66.72 | 37.12 |
| 4 | Wheat flour | Beijing | 265.28 | <LOD | 61.76 | 7.68 |
| 5 | Wheat flour | Henan | 265.28 | <LOD | 45.92 | 6.56 |
| 6 | Wheat flour | Henan | 258.40 | 19.20 | 60.00 | 9.92 |
| 7 | Wheat flour | Henan | 245.92 | <LOD | 75.52 | 9.28 |
| 8 | Wheat flour | Henan | 244.16 | 16.00 | 72.80 | 10.08 |
| 9 | Wheat flour | Henan | 231.36 | 17.12 | 62.24 | 14.72 |
| 10 | Wheat flour | Shandong | 228.16 | <LOD | 50.08 | 18.56 |
| 11 | Wheat flour | Beijing | 220.48 | <LOD | 46.56 | 4.16 |
| 12 | Wheat flour | Shandong | 217.76 | <LOD | 51.68 | 5.76 |
| 13 | Wheat flour | Henan | 200.32 | 16.64 | 82.08 | 4.48 |
| 14 | Wheat flour | Anhui | 193.44 | 23.68 | 40.48 | 20.48 |
| 15 | Wheat flour | Henan | 178.88 | <LOD | 47.20 | 6.40 |
| 16 | Wheat flour | Anhui | 171.36 | 24.16 | 70.72 | 11.52 |
| 17 | Wheat flour | Shandong | 168.80 | <LOD | 75.52 | 2.40 |
| 18 | Wheat flour | Beijing | 166.08 | <LOD | 47.52 | 1.44 |
| 19 | Wheat flour | Henan | 163.04 | <LOD | 35.68 | 2.56 |
| 20 | Wheat flour | Henan | 157.76 | <LOD | 29.44 | 3.84 |
| 21 | Wheat flour | Jilin | 154.88 | <LOD | 29.44 | 5.92 |
| 22 | Wheat flour | Beijing | 152.00 | <LOD | 42.24 | 11.52 |
| 23 | Wheat flour | Shandong | 147.36 | <LOD | 38.88 | 3.68 |
| 24 | Wheat flour | Beijing | 145.92 | <LOD | 27.84 | 12.64 |
| 25 | Wheat flour | Shandong | 145.44 | <LOD | 19.04 | 1.60 |
| 26 | Wheat flour | Anhui | 143.52 | <LOD | 45.44 | 7.04 |
| 27 | Wheat flour | Anhui | 141.28 | 30.08 | 74.08 | 13.76 |
| 28 | Wheat flour | Shandong | 140.80 | <LOD | 48.00 | 2.56 |
| 29 | Wheat flour | Anhui | 140.48 | <LOD | 39.20 | 5.28 |
| 30 | Wheat flour | Henan | 140.00 | <LOD | 40.16 | <LOD |
| 31 | Wheat flour | Henan | 136.32 | <LOD | 30.40 | 3.84 |
| 32 | Wheat flour | Shandong | 134.88 | <LOD | 32.16 | 2.72 |
| 33 | Wheat flour | Henan | 133.60 | <LOD | 25.12 | 3.04 |
| 34 | Wheat flour | Henan | 126.56 | <LOD | 44.48 | 2.88 |
| 35 | Wheat flour | Shandong | 126.08 | <LOD | 42.40 | 1.60 |
| 36 | Wheat flour | Beijing | 123.52 | <LOD | 36.80 | 6.24 |
| 37 | Wheat flour | Henan | 122.08 | <LOD | 27.04 | 3.20 |
| 38 | Wheat flour | Shandong | 116.96 | <LOD | 26.08 | 1.92 |
| 39 | Wheat flour | Beijing | 116.80 | <LOD | 35.36 | 5.60 |
| 40 | Wheat flour | Shandong | 116.00 | <LOD | 37.28 | 2.56 |
| 41 | Wheat flour | Henan | 115.84 | <LOD | 29.28 | 3.68 |
| 42 | Wheat flour | Shandong | 114.72 | <LOD | 22.88 | 1.76 |
| 43 | Wheat flour | Henan | 114.08 | <LOD | 35.52 | 4.00 |
| 44 | Wheat flour | Henan | 113.92 | <LOD | 33.60 | 6.24 |
| 45 | Wheat flour | Jilin | 113.92 | <LOD | 24.64 | 3.52 |
| 46 | Wheat flour | Shandong | 112.80 | <LOD | 35.36 | 2.40 |
| 47 | Wheat flour | Shandong | 109.28 | <LOD | 24.00 | 1.28 |
| 48 | Wheat flour | Beijing | 108.64 | <LOD | 27.20 | 4.32 |
| 49 | Wheat flour | Beijing | 108.48 | <LOD | 22.88 | 5.12 |
| 50 | Wheat flour | Henan | 107.36 | <LOD | 85.76 | 2.40 |
| 51 | Wheat flour | Henan | 106.56 | <LOD | 46.88 | 2.56 |
| 52 | Wheat flour | Henan | 106.24 | <LOD | 58.08 | 2.56 |
| 53 | Wheat flour | Jilin | 105.76 | <LOD | 15.68 | 18.88 |
| 54 | Wheat flour | Shandong | 105.60 | <LOD | 44.96 | 1.12 |
| 55 | Wheat flour | Henan | 104.48 | <LOD | 31.04 | 5.92 |
| 56 | Wheat flour | Shandong | 104.48 | <LOD | 24.96 | 1.12 |
| 57 | Wheat flour | Shandong | 104.00 | <LOD | 33.12 | 2.72 |
| 58 | Wheat flour | Anhui | 103.84 | <LOD | 26.24 | 5.44 |
| 59 | Wheat flour | Beijing | 99.84 | <LOD | 36.48 | 4.48 |
| 60 | Wheat flour | Henan | 99.68 | <LOD | 19.36 | 3.84 |
| 61 | Wheat flour | Henan | 99.36 | <LOD | 32.32 | 3.52 |
| 62 | Wheat flour | Henan | 97.76 | <LOD | 40.96 | 3.68 |
| 63 | Wheat flour | Shandong | 96.16 | <LOD | 16.00 | 1.76 |
| 64 | Wheat flour | Beijing | 95.68 | <LOD | 21.60 | 2.24 |
| 65 | Wheat flour | Henan | 94.72 | <LOD | 31.84 | 1.12 |
| 66 | Wheat flour | Jilin | 94.56 | <LOD | 128.64 | 0.32 |
| 67 | Wheat flour | Jilin | 94.24 | <LOD | 15.04 | 0.64 |
| 68 | Wheat flour | Shandong | 93.92 | <LOD | 16.64 | 1.12 |
| 69 | Wheat flour | Anhui | 93.28 | 24.00 | 27.84 | 10.40 |
| 70 | Wheat flour | Shandong | 92.48 | <LOD | 23.68 | 2.24 |
| 71 | Wheat flour | Anhui | 91.04 | <LOD | 18.08 | 3.52 |
| 72 | Wheat flour | Shandong | 90.88 | <LOD | 28.32 | 1.92 |
| 73 | Wheat flour | Henan | 90.40 | <LOD | 26.24 | 3.20 |
| 74 | Wheat flour | Shandong | 90.24 | <LOD | 25.76 | 2.72 |
| 75 | Wheat flour | Anhui | 88.80 | <LOD | 30.24 | 4.16 |
| 76 | Wheat flour | Anhui | 87.68 | 24.00 | 40.00 | 9.28 |
| 77 | Wheat flour | Shandong | 87.20 | <LOD | 26.72 | 0.80 |
| 78 | Wheat flour | Beijing | 86.88 | <LOD | 16.80 | 7.68 |
| 79 | Wheat flour | Henan | 86.88 | <LOD | 31.52 | 3.84 |
| 80 | Wheat flour | Anhui | 86.88 | <LOD | 39.84 | 4.00 |
| 81 | Wheat flour | Shandong | 85.12 | <LOD | 25.60 | 1.60 |
| 82 | Wheat flour | Henan | 84.32 | <LOD | 24.32 | 0.96 |
| 83 | Wheat flour | Henan | 83.20 | <LOD | 27.20 | 0.96 |
| 84 | Wheat flour | Beijing | 82.24 | <LOD | 18.72 | 2.72 |
| 85 | Wheat flour | Anhui | 79.36 | <LOD | 14.72 | 1.76 |
| 86 | Wheat flour | Anhui | 79.20 | <LOD | 21.12 | 3.04 |
| 87 | Wheat flour | Shandong | 79.04 | <LOD | 11.20 | 1.44 |
| 88 | Wheat flour | Shandong | 78.72 | <LOD | 19.68 | 3.52 |
| 89 | Wheat flour | Beijing | 78.24 | <LOD | 19.84 | 1.44 |
| 90 | Wheat flour | Shandong | 78.08 | <LOD | 16.80 | 1.28 |
| 91 | Wheat flour | Jilin | 77.92 | <LOD | 18.56 | 1.44 |
| 92 | Wheat flour | Jilin | 77.12 | <LOD | 11.52 | 1.92 |
| 93 | Wheat flour | Shandong | 76.64 | <LOD | 27.84 | 1.28 |
| 94 | Wheat flour | Anhui | 75.84 | <LOD | 43.52 | 1.44 |
| 95 | Wheat flour | Beijing | 73.92 | <LOD | 25.28 | 2.56 |
| 96 | Wheat flour | Anhui | 73.76 | <LOD | 23.68 | 5.60 |
| 97 | Wheat flour | Jilin | 72.48 | <LOD | 23.52 | 3.68 |
| 98 | Wheat flour | Shandong | 72.32 | <LOD | 17.12 | 1.28 |
| 99 | Wheat flour | Henan | 72.00 | <LOD | 29.92 | 2.40 |
| 100 | Wheat flour | Henan | 70.88 | <LOD | 25.44 | 2.40 |
| 101 | Wheat flour | Shandong | 70.24 | <LOD | 18.56 | 0.96 |
| 102 | Wheat flour | Shandong | 69.28 | <LOD | 20.32 | 2.40 |
| 103 | Wheat flour | Shandong | 66.40 | <LOD | 12.96 | 0.96 |
| 104 | Wheat flour | Jilin | 65.92 | <LOD | 12.32 | 1.60 |
| 105 | Wheat flour | Henan | 65.60 | <LOD | 28.48 | 1.44 |
| 106 | Wheat flour | Shandong | 64.32 | <LOD | 17.60 | 1.44 |
| 107 | Wheat flour | Shandong | 63.68 | <LOD | 32.96 | 0.64 |
| 108 | Wheat flour | Shandong | 63.04 | <LOD | 18.40 | 1.76 |
| 109 | Wheat flour | Anhui | 62.24 | <LOD | 10.72 | 1.92 |
| 110 | Wheat flour | Anhui | 61.60 | <LOD | 11.20 | 0.64 |
| 111 | Wheat flour | Anhui | 60.48 | <LOD | 21.92 | 0.80 |
| 112 | Wheat flour | Shandong | 59.68 | <LOD | 17.60 | 2.08 |
| 113 | Wheat flour | Shandong | 58.56 | <LOD | 23.84 | 1.28 |
| 114 | Wheat flour | Shandong | 58.56 | <LOD | 28.64 | 1.28 |
| 115 | Wheat flour | Shandong | 57.60 | <LOD | 14.40 | 0.48 |
| 116 | Wheat flour | Shandong | 57.44 | <LOD | 14.08 | 0.80 |
| 117 | Wheat flour | Shandong | 57.12 | <LOD | 15.68 | 0.96 |
| 118 | Wheat flour | Shandong | 56.96 | <LOD | 16.16 | 0.80 |
| 119 | Wheat flour | Henan | 56.48 | <LOD | 22.72 | 0.80 |
| 120 | Wheat flour | Jilin | 56.32 | <LOD | 18.40 | 0.80 |
| 121 | Wheat flour | Anhui | 55.84 | <LOD | 13.28 | 2.40 |
| 122 | Wheat flour | Jilin | 55.84 | <LOD | 28.32 | 2.08 |
| 123 | Wheat flour | Henan | 51.84 | <LOD | 20.64 | 1.28 |
| 124 | Wheat flour | Henan | 51.20 | <LOD | 16.96 | 1.44 |
| 125 | Wheat flour | Jilin | 50.72 | <LOD | 15.52 | 2.88 |
| 126 | Wheat flour | Shandong | 50.40 | <LOD | 14.88 | 0.32 |
| 127 | Wheat flour | Henan | 49.60 | <LOD | 33.76 | <LOD |
| 128 | Wheat flour | Beijing | 48.96 | <LOD | 21.12 | 2.56 |
| 129 | Wheat flour | Shandong | 48.96 | <LOD | 15.36 | 0.64 |
| 130 | Wheat flour | Anhui | 47.84 | <LOD | 23.36 | 0.80 |
| 131 | Wheat flour | Shandong | 47.36 | <LOD | 5.28 | 1.92 |
| 132 | Wheat flour | Shandong | 45.92 | <LOD | 8.00 | 0.80 |
| 133 | Wheat flour | Shandong | 45.92 | <LOD | 42.24 | 2.40 |
| 134 | Wheat flour | Shandong | 45.60 | <LOD | 11.84 | 0.64 |
| 135 | Wheat flour | Shandong | 44.16 | <LOD | 26.56 | 0.64 |
| 136 | Wheat flour | Anhui | 43.52 | <LOD | 13.12 | 0.80 |
| 137 | Wheat flour | Shandong | 43.20 | <LOD | 14.72 | 0.80 |
| 138 | Wheat flour | Jilin | 43.04 | <LOD | 5.12 | <LOD |
| 139 | Wheat flour | Henan | 42.40 | <LOD | 16.48 | 1.76 |
| 140 | Wheat flour | Henan | 42.08 | <LOD | 15.84 | 1.12 |
| 141 | Wheat flour | Jilin | 41.60 | <LOD | 19.68 | 1.76 |
| 142 | Wheat flour | Jilin | 41.44 | <LOD | 11.04 | <LOD |
| 143 | Wheat flour | Jilin | 41.12 | <LOD | 7.20 | 0.96 |
| 144 | Wheat flour | Beijing | 39.52 | <LOD | 8.80 | 1.92 |
| 145 | Wheat flour | Shandong | 37.44 | <LOD | 15.84 | 0.48 |
| 146 | Wheat flour | Beijing | 37.28 | <LOD | 14.72 | 1.44 |
| 147 | Wheat flour | Shandong | 35.52 | <LOD | 9.12 | 0.48 |
| 148 | Wheat flour | Shandong | 35.04 | <LOD | 13.12 | 0.64 |
| 149 | Wheat flour | Shandong | 35.04 | <LOD | 13.44 | 1.44 |
| 150 | Wheat flour | Jilin | 34.40 | <LOD | 12.00 | 0.96 |
| 151 | Wheat flour | Henan | 32.80 | <LOD | 18.56 | <LOD |
| 152 | Wheat flour | Beijing | 32.64 | <LOD | 8.96 | 0.96 |
| 153 | Wheat flour | Henan | 31.68 | <LOD | 4.64 | 1.28 |
| 154 | Wheat flour | Shandong | 31.20 | <LOD | 12.48 | 0.80 |
| 155 | Wheat flour | Henan | 30.88 | <LOD | 9.92 | 15.68 |
| 156 | Wheat flour | Jilin | 30.72 | <LOD | 6.88 | <LOD |
| 157 | Wheat flour | Jilin | 30.56 | <LOD | 9.44 | 0.64 |
| 158 | Wheat flour | Beijing | 30.08 | <LOD | 6.24 | 1.76 |
| 159 | Wheat flour | Shandong | 29.28 | <LOD | 7.04 | 0.64 |
| 160 | Wheat flour | Jilin | 28.16 | <LOD | 30.24 | 0.96 |
| 161 | Wheat flour | Shandong | 28.00 | <LOD | 7.36 | 1.44 |
| 162 | Wheat flour | Jilin | 27.04 | <LOD | 9.44 | 0.48 |
| 163 | Wheat flour | Shandong | 26.72 | <LOD | 21.60 | 1.44 |
| 164 | Wheat flour | Shandong | 25.76 | <LOD | 6.88 | 1.28 |
| 165 | Wheat flour | Jilin | 25.44 | <LOD | 23.84 | <LOD |
| 166 | Wheat flour | Beijing | 24.32 | <LOD | 7.36 | 0.48 |
| 167 | Wheat flour | Shandong | 24.00 | <LOD | 9.76 | 0.96 |
| 168 | Wheat flour | Jilin | 23.52 | <LOD | 8.96 | <LOD |
| 169 | Wheat flour | Jilin | 22.08 | <LOD | 18.40 | 0.64 |
| 170 | Wheat flour | Shandong | 21.44 | <LOD | 7.84 | <LOD |
| 171 | Wheat flour | Beijing | 20.64 | <LOD | 6.40 | 0.48 |
| 172 | Wheat flour | Jilin | 17.44 | <LOD | 22.56 | <LOD |
| 173 | Wheat flour | Henan | 12.80 | <LOD | 4.64 | <LOD |
| 174 | Wheat flour | Beijing | 12.00 | <LOD | 4.64 | <LOD |
| 175 | Wheat flour | Beijing | 11.68 | <LOD | 6.24 | 0.48 |
| 176 | Wheat flour | Jilin | 9.44 | <LOD | 2.72 | 0.32 |
| 177 | Wheat flour | Henan | 8.96 | <LOD | 0.00 | <LOD |
| 178 | Wheat flour | Beijing | 6.08 | <LOD | 0.00 | 0.32 |
| 179 | Wheat flour | Jilin | 3.36 | <LOD | 2.72 | <LOD |
| 180 | Wheat flour | Anhui | 2.40 | <LOD | 0.00 | <LOD |
| 181 | Wheat flour | Jilin | 1.76 | <LOD | 0.00 | 0.48 |
| 182 | dried noodle | Beijing | <LOD | <LOD | <LOD | <LOD |
| 183 | dried noodle | Beijing | <LOD | <LOD | <LOD | <LOD |
| 184 | dried noodle | Beijing | 157.60 | 11.78 | 29.88 | 1.12 |
| 185 | dried noodle | Beijing | 150.34 | <LOD | 32.26 | 2.63 |
| 186 | dried noodle | Beijing | 150.34 | <LOD | 31.50 | 2.33 |
| 187 | dried noodle | Beijing | 131.93 | <LOD | 29.84 | 1.83 |
| 188 | dried noodle | Beijing | 113.77 | <LOD | 28.02 | 2.36 |
| 189 | dried noodle | Beijing | 89.11 | <LOD | 29.21 | 1.43 |
| 190 | dried noodle | Beijing | 87.81 | <LOD | 21.59 | 1.99 |
| 191 | dried noodle | Beijing | 82.98 | 10.69 | 18.00 | 1.86 |
| 192 | dried noodle | Beijing | 81.50 | <LOD | 18.29 | 2.67 |
| 193 | dried noodle | Beijing | 75.69 | <LOD | 21.47 | 0.18 |
| 194 | dried noodle | Beijing | 74.50 | <LOD | 19.71 | 0.24 |
| 195 | dried noodle | Beijing | 73.14 | <LOD | 20.56 | 0.61 |
| 196 | dried noodle | Beijing | 69.75 | <LOD | 18.04 | 4.10 |
| 197 | dried noodle | Beijing | 68.26 | <LOD | 27.31 | 0.23 |
| 198 | dried noodle | Beijing | 63.61 | <LOD | 15.96 | 3.69 |
| 199 | dried noodle | Beijing | 51.72 | <LOD | 11.75 | 1.54 |
| 200 | dried noodle | Beijing | 51.62 | <LOD | 13.73 | <LOD |
| 201 | dried noodle | Beijing | 51.47 | <LOD | 16.68 | 1.72 |
| 202 | dried noodle | Beijing | 46.98 | <LOD | 13.13 | 0.82 |
| 203 | dried noodle | Beijing | 43.44 | <LOD | 11.57 | <LOD |
| 204 | dried noodle | Beijing | 40.05 | <LOD | 13.80 | 0.23 |
| 205 | dried noodle | Beijing | 38.81 | <LOD | 14.94 | 0.58 |
| 206 | dried noodle | Beijing | 34.17 | <LOD | 12.57 | 0.27 |
| 207 | dried noodle | Beijing | 34.10 | 9.59 | 12.25 | 0.65 |
| 208 | dried noodle | Beijing | 34.05 | <LOD | 6.69 | 0.38 |
| 209 | dried noodle | Beijing | 31.79 | <LOD | 14.93 | <LOD |
| 210 | dried noodle | Beijing | 30.69 | <LOD | 13.71 | <LOD |
| 211 | dried noodle | Beijing | 30.57 | <LOD | 13.54 | <LOD |
| 212 | dried noodle | Beijing | 29.82 | <LOD | 7.43 | <LOD |
| 213 | dried noodle | Beijing | 28.60 | <LOD | 9.31 | 0.45 |
| 214 | dried noodle | Beijing | 27.37 | <LOD | 9.17 | <LOD |
| 215 | dried noodle | Beijing | 26.01 | <LOD | 5.56 | 0.20 |
| 216 | dried noodle | Beijing | 22.98 | <LOD | 7.17 | <LOD |
| 217 | dried noodle | Beijing | 22.93 | <LOD | 10.09 | 0.37 |
| 218 | dried noodle | Beijing | 21.85 | <LOD | 3.06 | 0.27 |
| 219 | dried noodle | Beijing | 21.31 | <LOD | 6.07 | 0.82 |
| 220 | dried noodle | Beijing | 21.08 | <LOD | 6.70 | 0.21 |
| 221 | dried noodle | Beijing | 20.95 | <LOD | <LOD | <LOD |
| 222 | dried noodle | Beijing | 20.13 | <LOD | 7.13 | 0.56 |
| 223 | dried noodle | Beijing | 19.91 | <LOD | 8.87 | <LOD |
| 224 | dried noodle | Beijing | 18.23 | <LOD | 5.20 | <LOD |
| 225 | dried noodle | Beijing | 16.36 | <LOD | 2.25 | <LOD |
| 226 | dried noodle | Beijing | 15.09 | <LOD | 5.58 | <LOD |
| 227 | dried noodle | Beijing | 13.82 | <LOD | 5.37 | <LOD |
| 228 | dried noodle | Beijing | 12.97 | <LOD | 3.33 | 0.79 |
| 229 | dried noodle | Beijing | 7.49 | <LOD | 2.91 | <LOD |
| 230 | dried noodle | Beijing | 7.45 | <LOD | <LOD | <LOD |
| 231 | dried noodle | Beijing | 7.02 | <LOD | <LOD | 0.19 |
| 232 | dried noodle | Beijing | 6.28 | <LOD | 3.89 | <LOD |
| 233 | dried noodle | Beijing | 4.86 | <LOD | <LOD | <LOD |
| 234 | bread | Beijing | <LOD | <LOD | <LOD | <LOD |
| 235 | bread | Beijing | 38.18 | <LOD | 21.80 | 0.18 |
| 236 | bread | Beijing | 35.97 | <LOD | 9.97 | <LOD |
| 237 | bread | Beijing | 32.92 | <LOD | 11.85 | 2.63 |
| 238 | bread | Beijing | 31.90 | <LOD | 27.19 | 0.98 |
| 239 | bread | Beijing | 31.74 | <LOD | 17.69 | 0.23 |
| 240 | bread | Beijing | 25.40 | <LOD | 11.30 | 1.99 |
| 241 | bread | Beijing | 19.31 | <LOD | 8.69 | 2.13 |
| 242 | bread | Beijing | 18.41 | <LOD | 9.28 | 6.49 |
| 243 | bread | Beijing | 17.86 | <LOD | 12.82 | 0.75 |
| 244 | bread | Beijing | 17.03 | <LOD | 7.93 | 0.96 |
| 245 | bread | Beijing | 15.69 | <LOD | 7.82 | 0.24 |
| 246 | bread | Beijing | 15.26 | <LOD | 10.48 | <LOD |
| 247 | bread | Beijing | 14.90 | <LOD | 10.38 | 0.39 |
| 248 | bread | Beijing | 14.38 | <LOD | 7.71 | <LOD |
| 249 | bread | Beijing | 13.89 | <LOD | 7.92 | 1.11 |
| 250 | bread | Beijing | 13.47 | <LOD | 7.26 | 0.43 |
| 251 | bread | Beijing | 11.69 | <LOD | 6.96 | <LOD |
| 252 | bread | Beijing | 10.61 | <LOD | 5.51 | <LOD |
| 253 | bread | Beijing | 10.32 | <LOD | 7.59 | 0.70 |
| 254 | bread | Beijing | 10.23 | <LOD | 9.61 | 0.31 |
| 255 | bread | Beijing | 9.45 | <LOD | 8.30 | <LOD |
| 256 | bread | Beijing | 9.26 | <LOD | 5.53 | <LOD |
| 257 | bread | Beijing | 8.82 | <LOD | 4.97 | 0.33 |
| 258 | bread | Beijing | 8.81 | <LOD | 9.79 | <LOD |
| 259 | bread | Beijing | 8.81 | <LOD | 11.24 | 0.97 |
| 260 | bread | Beijing | 8.59 | <LOD | 7.08 | <LOD |
| 261 | bread | Beijing | 8.55 | <LOD | 8.34 | <LOD |
| 262 | bread | Beijing | 8.53 | 9.98 | 3.87 | 1.30 |
| 263 | bread | Beijing | 7.94 | <LOD | 9.15 | 0.40 |
| 264 | bread | Beijing | 7.83 | <LOD | 3.82 | 0.42 |
| 265 | bread | Beijing | 7.64 | <LOD | 3.20 | <LOD |
| 266 | bread | Beijing | 7.52 | <LOD | 3.13 | 0.32 |
| 267 | bread | Beijing | 7.13 | <LOD | 4.53 | <LOD |
| 268 | bread | Beijing | 6.52 | <LOD | 6.04 | <LOD |
| 269 | bread | Beijing | 6.51 | <LOD | 7.48 | <LOD |
| 270 | bread | Beijing | 6.12 | <LOD | 5.03 | <LOD |
| 271 | bread | Beijing | 5.37 | <LOD | <LOD | <LOD |
| 272 | bread | Beijing | 4.94 | <LOD | 5.00 | <LOD |
| 273 | bread | Beijing | 4.87 | <LOD | 5.26 | <LOD |
| 274 | bread | Beijing | 3.93 | <LOD | <LOD | <LOD |
| 275 | bread | Beijing | 3.82 | <LOD | <LOD | <LOD |
| 276 | bread | Beijing | 3.67 | <LOD | 4.25 | <LOD |
| 277 | bread | Beijing | 3.39 | <LOD | <LOD | <LOD |
| 278 | bread | Beijing | 3.26 | <LOD | 3.56 | <LOD |
| 279 | bread | Beijing | 3.24 | <LOD | <LOD | <LOD |
| 280 | bread | Beijing | 3.17 | <LOD | 3.68 | <LOD |
| 281 | bread | Beijing | 2.90 | <LOD | <LOD | <LOD |
| 282 | bread | Beijing | 2.25 | <LOD | <LOD | <LOD |
| 283 | bread | Beijing | 1.95 | <LOD | <LOD | 0.19 |
| 284 | steamed bread | Beijing | 46.30 | <LOD | 26.11 | <LOD |
| 285 | steamed bread | Beijing | 38.27 | <LOD | 14.91 | <LOD |
| 286 | steamed bread | Beijing | 37.62 | <LOD | 31.58 | 0.31 |
| 287 | steamed bread | Beijing | 37.53 | <LOD | 14.01 | 0.38 |
| 288 | steamed bread | Beijing | 37.29 | <LOD | 26.02 | 0.26 |
| 289 | steamed bread | Beijing | 35.86 | <LOD | 25.02 | 0.34 |
| 290 | steamed bread | Beijing | 29.06 | <LOD | 18.92 | 0.65 |
| 291 | steamed bread | Beijing | 28.97 | <LOD | 29.22 | 0.43 |
| 292 | steamed bread | Beijing | 28.94 | <LOD | 8.12 | <LOD |
| 293 | steamed bread | Beijing | 26.92 | <LOD | 19.25 | 0.59 |
| 294 | steamed bread | Beijing | 26.37 | <LOD | 11.18 | 1.39 |
| 295 | steamed bread | Beijing | 25.79 | <LOD | 24.26 | 0.63 |
| 296 | steamed bread | Beijing | 25.71 | <LOD | 10.52 | 0.29 |
| 297 | steamed bread | Beijing | 25.33 | <LOD | 14.91 | 0.21 |
| 298 | steamed bread | Beijing | 24.64 | <LOD | 9.34 | 0.41 |
| 299 | steamed bread | Beijing | 23.20 | <LOD | 10.32 | 1.24 |
| 300 | steamed bread | Beijing | 22.33 | 5.55 | 9.49 | 1.41 |
| 301 | steamed bread | Beijing | 21.69 | <LOD | 13.28 | 1.12 |
| 302 | steamed bread | Beijing | 21.06 | <LOD | 9.13 | 0.81 |
| 303 | steamed bread | Beijing | 19.77 | <LOD | 7.17 | 0.66 |
| 304 | steamed bread | Beijing | 19.28 | <LOD | 5.45 | 0.77 |
| 305 | steamed bread | Beijing | 16.76 | <LOD | 3.54 | 0.82 |
| 306 | steamed bread | Beijing | 16.70 | <LOD | 5.24 | 0.67 |
| 307 | steamed bread | Beijing | 15.98 | 5.12 | 6.73 | 0.64 |
| 308 | steamed bread | Beijing | 15.68 | <LOD | 7.06 | 0.65 |
| 309 | steamed bread | Beijing | 14.56 | 7.37 | 4.77 | 1.00 |
| 310 | steamed bread | Beijing | 14.27 | <LOD | 4.24 | 0.60 |
| 311 | steamed bread | Beijing | 14.08 | <LOD | 3.45 | 0.56 |
| 312 | steamed bread | Beijing | 14.05 | <LOD | 5.71 | <LOD |
| 313 | steamed bread | Beijing | 13.54 | <LOD | 4.31 | <LOD |
| 314 | steamed bread | Beijing | 13.35 | <LOD | 6.02 | 0.61 |
| 315 | steamed bread | Beijing | 13.24 | <LOD | 3.02 | 0.61 |
| 316 | steamed bread | Beijing | 12.76 | <LOD | 2.92 | <LOD |
| 317 | steamed bread | Beijing | 12.45 | <LOD | 7.76 | 0.24 |
| 318 | steamed bread | Beijing | 12.42 | <LOD | 4.83 | <LOD |
| 319 | steamed bread | Beijing | 11.59 | <LOD | 2.46 | 0.34 |
| 320 | steamed bread | Beijing | 11.15 | <LOD | 5.69 | <LOD |
| 321 | steamed bread | Beijing | 10.22 | 6.16 | 2.58 | <LOD |
| 322 | steamed bread | Beijing | 8.58 | <LOD | 4.79 | <LOD |
| 323 | steamed bread | Beijing | 6.56 | <LOD | 6.06 | <LOD |
